# Epidemiology, health policy and public health implications of visual impairment and age-related eye diseases in mainland China

**DOI:** 10.3389/fpubh.2022.966006

**Published:** 2022-11-09

**Authors:** Cong Li, Bo Zhu, Jie Zhang, Peng Guan, Guisen Zhang, Honghua Yu, Xiaohong Yang, Lei Liu

**Affiliations:** ^1^Department of Ophthalmology, Guangdong Eye Institute, Guangdong Provincial People's Hospital, Guangdong Academy of Medical Sciences, Guangzhou, China; ^2^School of Medicine, South China University of Technology, Guangzhou, China; ^3^Cancer Hospital of China Medical University/Liaoning Cancer Hospital & Institute, Shenyang, China; ^4^Department of Retina, Weifang Eye Hospital, Weifang, China; ^5^Department of Epidemiology, School of Public Health, China Medical University, Shenyang, China; ^6^Department of Retina, Inner Mongolia Chaoju Eye Hospital, Hohhot, China

**Keywords:** age-related eye diseases, epidemiology, prevalence, incidence, risk factors

## Abstract

The prevalence of visual impairment (VI) and age-related eye diseases has increased dramatically with the growing aging population in mainland China. However, there is limited comprehensive evidence on the progress of ophthalmic epidemiological research in mainland China to enhance our awareness of the prevention of eye diseases to inform public health policy. Here, we conducted a literature review of the population-based epidemiology of VI and age-related eye diseases in mainland China from the 1st of January 1946 to the 20th of October 2021. No language restrictions were applied. There was significant demographic and geographic variation in the epidemic of VI and age-related eye diseases. There are several factors known to be correlated to VI and age-related eye diseases, including age, gender, family history, lifestyle, biological factors, and environmental exposures; however, evidence relating to genetic predisposition remains unclear. In addition, posterior segment eye diseases, including age-related macular degeneration and diabetic retinopathy, are amongst the major causes of irreversible visual impairments in the senile Chinese population. There remains a significant prevention gap, with only a few individuals showing awareness and achieving optimal medical care with regards to age-related eye diseases. Multiple challenges and obstacles need to be overcome, including the accelerated aging of the Chinese population, the lack of structured care delivery in many underdeveloped regions, and unequal access to care. Despite the progress to date, there are few well-conducted multi-center population-based studies following a single protocol in mainland China, which findings can hopefully provide valuable cues for governmental decision-making and assist in addressing and halting the incidence of VI and age-related eye diseases in China.

## Introduction

Age-related eye diseases, including senile cataract, glaucoma, diabetic retinopathy (DR), and age-related macular degeneration (AMD) are the leading causes of vision loss in the elderly worldwide (1). Mainland China comprises one-fifth of the world's population with 1.41 billion people, including 190 million (13.5%) individuals aged 65 years and above. Furthermore, a substantial increase in the number of older persons is expected in the next few decades (http://www.stats.gov.cn/tjsj/tjgb/rkpcgb/). The increasing aged population has brought about a rising trend in the prevalence and incidence of age-related eye diseases, and the government of mainland China has been making significant efforts toward reducing the prevalence of eye diseases, such as a gradual increase in research funding (2). According to the Global Burden of Diseases, Injuries, and Risk Factors Study (GBD) 2019, the number of individuals with blindness among the Chinese population is exhibiting an upwards trend, rising from 5.29 million in 1990 to 8.69 million in 2019, thus contributing to the global disease burden (3). Several large single-center population-based epidemiological studies have investigated eye diseases among middle-aged and elderly Chinese adults (4–7). Furthermore, there have been several meta-analyses evaluating the prevalence of age-related eye diseases among the Chinese population in the past decades (8–12). According to the advances made in epidemiological and evidence-based studies on age-related eye diseases, great achievements have been made in the areas of eye health management, public health, and health economics. Furthermore, many new and ongoing studies have been conducted over recent years, which have provided information and interesting patterns relating to age-related eye diseases in mainland China. These previous findings imply that an update concerning the health care of patients with age-related eye diseases is still necessary (4, 13, 14). Furthermore, understanding this epidemic evidence may provide invaluable insights into the progress of ophthalmic epidemiological research in mainland China which will hopefully be instrumental for public health and health services. In this literature review, we aimed to summarize the current progress of ophthalmic epidemiological research in mainland China, thus enhancing awareness for the need to prevent eye diseases and informing public health policy.

## Search strategy and selection criteria

The literature selected for this review was sourced from Embase, PubMed/MEDLINE, Cumulative Index of Nursing and Allied Health Literature (CINAHL), Cochrane Library, and three Chinese databases [Wanfang, China National Knowledge Infrastructure (CNKI), and SinoMed] from the 1st of January 1946 to the 20th of October 2021, and included over 300 articles from existing literature. Searches were performed using the keywords “epidemiology” AND “ophthalmology” AND “population-based study” AND “China.” No language restrictions were applied.

We used the following criteria for the inclusion of articles. First, articles needed to provide epidemic information relating to the prevalence, incidence rates, or associated factors of age-related eye diseases (e.g., visual impairment, cataract, glaucoma, DR, or AMD). Second, epidemic information needed to include middle-aged and participants above 50 years-of-age. The middle-aged group consisted of individuals aged 50–65 years, a period in which many chronic physical conditions start to develop. Third, the study needed to be population-based in the general population and/or a primary care setting; the sample size needed to be larger than 1,000. Fourth, the study needed to have been conducted in mainland China.

Searches were performed independently by both CL and LL; then, the results were compared and discussed. Multiple publications from the same study were compared and the most updated or complete studies were retained. Studies that reported sub-center information embedded within a total multicenter data were excluded. In cases of disagreement, XHY was consulted for a final decision.

## Visual impairment in mainland China

The Vision Loss Expert Group of the Global Burden of Disease Study previously estimated that visual impairment (VI) affects ~10% of individuals aged 50 years or older in China (15). There are two definitions of VI and blindness; these include criteria from the World Health Organization (WHO) and United States (US). According to WHO criteria, VI is defined as a Snellen visual acuity (VA) < 20/60 in the better-seeing eye, and blindness as a VA < 20/400 in the better-seeing eye. Specifically, the classification of VI severity recommended by the Resolution of the International Council of Ophthalmology and WHO Consultation includes severe visual impairment (SVI) as a VA of 3/60 or better and <6/60, moderate visual impairment (MVI) as a VA of 6/60 or better and <6/18, and early visual impairment (EVI) as a VA of 6/18 to <6/12 (16). For the US criteria, VI is defined as a VA < 20/40 in the better-seeing eye, and blindness as a VA < 20/200 in the better-seeing eye (17). There is known variability in the etiologies included in the definition of VI; this heterogeneity can influence the incidence and prevalence rates. The leading causes of VI have been reported to be cataract, uncorrected refractive error and posterior segment disorders including age-related macular degeneration, myopic macular degeneration, diabetic retinopathy, and other optic nerve atrophy (16, 18). Twenty-seven articles were reviewed in total; refractive error constituted a VI factor in 25 studies, cataract in 23 studies, posterior segment disorders in 17 studies and glaucoma in 9 studies, respectively.

### Prevalence

**Figure 1:**
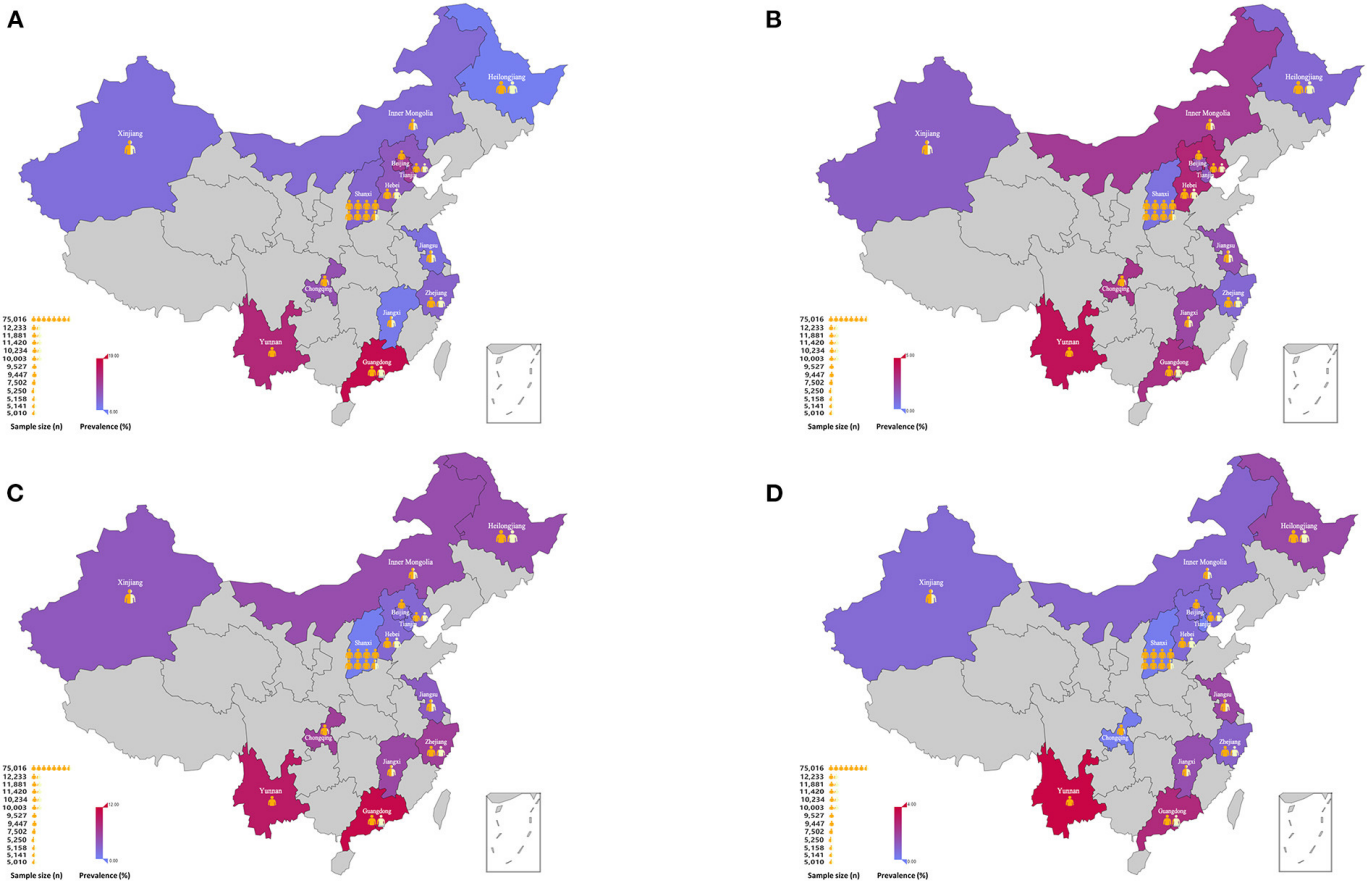
Geographical distribution of population-based studies in prevalence of low vision and blindness using the WHO criteria in mainland China. **(A)** the prevalence of low vision based on PVA; **(B)** the prevalence of blindness based on PVA; **(C)** the prevalence of low vision based on BCVA; **(D)** the prevalence of blindness based on BCVA. WHO, World Health Organization; PVA, presenting visual acuity; BCVA, best corrected visual acuity.

The characteristics of population-based studies relating to the prevalence of VI in mainland China have been described in Supplementary Table S1. Generally, the prevalence of VI (both low vision and blindness) appeared to vary across different regions in mainland China (Figure 1). The prevalence of VI also varies when applying different criteria. Using the WHO criteria, the prevalence of low vision and blindness based on a presenting visual acuity (PVA) was 0.77–25.53% and 0.6–3.7% (16, 19, 20); the prevalence based on best corrected visual acuity (BCVA) was 0.43–13.79% and 0.11–2.1% among adults aged 40 years and older, respectively (20–24). However, when applying the US criteria, the prevalence of low vision and blindness by PVA was 9.5–23.8%, and 1.2–1.9% (23–25), respectively; the prevalence by BCVA was 2.9–13.7% and 0.6–4.2%, respectively (23, 24). The prevalence rates for both low vision and blindness were lower in BCVA than those by PVA. However, PVA is always applied in population-based studies as it better reflects normal daily functional requirements when compared to uncorrected VA or BCVA. These findings reflect that there is a need for vision correction in Chinese adults.

#### Temporal trends

To date, there have been two multicenter population-based investigations on VI. In 2006, the second China National Sample Survey Disability (CNSSD) was conducted and involved 2.6 million samples aged 18 years and older from 5,964 communities/areas, 2,980 towns/townships and 734 counties of 31 provinces. This study found that the weighted prevalence of low vision and blindness was 1.1%, and 0.58% when applying the WHO criteria based on BCVA, respectively (26). In the same year, the China Nine-Province Survey (CNPS), including 45,747 adults above 50 years of age, found that with BCVA, the prevalence of VI was 5.30 and 1.93% for blindness, respectively (27). These differences can largely be attributed to those participants in the CNPS being relatively older. According to a follow-up of the CNPS in 2014, the prevalence of presenting vision impairment blindness (using the US criteria) would have decreased to 1.66% from 2.29% in 2006 (28). Similarly, in the current review, we observed a decreasing temporal trend in the prevalence of blindness (using the WHO definition and based on BCVA) over the past decades, from 3.19 to 1.20%. This might reflect the improvement of the health care system in mainland China over the past 25 years. Due to the improvement of the healthcare system in some areas of China, and a better awareness of ocular diseases, such as cataract and glaucoma, which contribute to the main bulk of VI, these diseases are being detected and managed earlier, with less likelihood for them causing visual loss. In 1999, the WHO launched the Global Initiative for the Elimination of Avoidable Blindness; these strategy is known as “Vision 2020: the Right to Sight” and “Sight first, China action” in China (29). In addition, the Chinese Thirteenth Five-Year Plan for Eye Health has promoted eye care services for Chinese residents. However, with China's rapid socioeconomic development and the intensification of population aging, VI is still a significant health problem for the Chinese population.

#### Regional disparity

When considering specific geographical area, most population-based studies were conducted in rural and Eastern China. The prevalence of VI among the rural parts of China was higher than in urban areas (26, 30). Furthermore, the blindness by PVA (using the WHO definition) occurred more frequently among the population living in western China. The estimates obtained for blindness (3.7%) in Kunming, Yunnan were higher than those reported in previous surveys carried out in Eastern China such as Shunyi county (2.8% in individuals ≥ 50 years; PVA < 6/60) (31), and Doumen county (2.7% in individuals ≥ 50 years; PVA < 3/60) (20). Notably, the prevalence of blindness in the Kandze Prefecture (1.59%, Sichuan Province, western China) (16) resembled findings from rapid assessments of avoidable blindness (RAAB) studies in Jiangxi Province (1.5–1.8%) (32) and Chaonan Region (2.4%, Guangdong Province, eastern China) (33), but was lower than that in Hainan Province (3.5%) (34). One possible reason for the wide geographic variations could be the urban-rural disparities and their associated socioeconomic status.

#### Ethnic disparity

So far, the epidemiological investigation of VI in ethnic minority populations is very limited in mainland China. Two previous studies showed that the prevalence of VI among middle-aged and elder Dai (15.9%) and Bai (18.91%) individuals in Yunnan was higher than in Han Chinese (10%) when applying the WHO definition and based on PVA (23, 35). Therefore, health care accessibility among ethnic minority populations cannot be ignored. Further efforts and resources should be applied to prevent age-related eye diseases of minorities with VI.

### Incidence

The characteristics of population-based studies relating to the incidence of VI incidence in mainland China are described in Supplementary Table S2. To date, few population-based studies have collected prospective data on their original cohorts; therefore scarce information exists on the incidence of VI in mainland China. Generally, the overall 5-year incidence rate of low vision and blindness (using the WHO definition and based on PVA) was 1.7–12.4% and 0.33%; however, the rate was 0.5–5.38% and 0.x1–0.33% according to BCVA, respectively (36, 37). Notably, this rate increased to 22.9 and 9.43% after 10 years follow-up in the Liwan eye study (38). The proportion of the Chinese population aged 60 years or older is projected to grow from 15% in 2015 to 35% by 2050 (https://population.un.org/wpp/[EB/OL]). Because the risk of VI increases sharply with age, the burden of VI in China will therefore rise dramatically in the next few decades. Although many public health policies to expand the delivery of ophthalmic services throughout China have been implemented over recent years, further efforts need to focus on increasing accessibility and the affordability of eye care in rural regions such as Handan, Hebei province.

Geographically, the 5-year incidence of low vision was higher in Liwan than in Handan and Beijing (12.4 vs. 4.3 and 1.7%) (36, 37, 39). The differences of incidence rates between these areas were surprising given that participants in Liwan were urban residents; in contrast, the Handan sample included rural residents while the Beijing eye study included a mixture of urban and rural dwellers in northern China. The comparison of incidence estimates between different studies should account for methodological disparities such as definitions of VI, age ranges of the study subjects (Handan ≥ 30 years, Beijing ≥ 40 years, and Liwan ≥ 50 years) and differences in environmental factors and healthcare service delivery differences.

### The main factors associated with visual impairment

Several key factors have been correlated with VI, including age, gender, education, and socioeconomic development. The prevalence and incidence of VI has increased significantly with population aging. Given the accelerated population aging in mainland China, it is vital to provide better eye care and vision health policy for the elderly. Because of the different anatomical, endocrine, and social features between males and females, it has been established that females are associated with a higher prevalence of low vision and blindness. In addition, a population with higher levels of education and income would have improved access to better health care services, thus reducing the prevalence of VI. These findings highlight the importance of vision health management among particular populations such as older adults, females and those with a low education level or a low income. However, many other associated factors (such as genetic predisposition) are still unclear.

#### Age

The numbers of individuals with low vision and blindness are likely going to increase as the population of China ages rapidly (40). The significant association of age with the incidence and prevalence of low vision and blindness in mainland China is similar to that of other countries (41–43), and is of prime importance to program planners given the aging population. As expected, age is one of the most important predictors of VI in the Chinese population. Better eye care services would help care providers, policy makers, and health agencies to plan their activities to control VI among the Chinese population.

#### Gender

In the current review, the general prevalence of low vision and blindness among females was much higher than that of males, and this was in accordance with a recent meta-analysis (females: 15.6% for PVA, 7.1% for BCVA; males: 12.3% for PVA, 5.6% for BCVA) (44). Anatomical and endocrine features might contribute to the gender difference associated with the prevalence of age-related eye disease prevalence, as females have proven to be at a higher risk of developing primary angle-closure glaucoma (PACG), one of the most common causes of irreversible blindness globally (45, 46). The Chinese government is constantly improving the social status of women, although in some rural areas, the social status of females is relatively lower with few health care services, thus leading to a higher prevalence of VI. However, among ethnic Dai adults aged 50 years or older in a rural community in Xishuangbanna Autonomous Prefecture, Yunnan province, male residents were shown to be more likely to be affected by low vision but less likely to be blind when compared with females (23). Moreover, there was no significant association between both in the incidence of low vision and blindness incident and gender in Liwan and Handan (36, 39). However, the 5-year incidence of VI (using the WHO definition and based on BCVA) was significantly higher in females than in males (0.8 vs. 0.2%) (37). There is a need to conduct a population-based and prospective national wide survey on VI to investigate geographical variations in gender difference, including confounding factors such as the accessibility or use of eye-care services, culture and socioeconomic development (47).

#### Education and socioeconomic development

It is important to highlight the significant difference in the prevalence of VI according to different educational levels or income groups. According to cross-sectional and cohort studies, individuals with higher levels of education and income were associated with a lower likelihood of VI than those with illiteracy (0 vs. 2.5%) or lower income (0.2 vs. 0.9%) in mainland China (37, 48). Individuals with a higher education and associated higher income should have access to better health care services. Highly educated people are also more likely to be better informed and make better decisions when it comes to health-related problems. However, VI was not associated with income levels after the best correction of vision (49), which might mean that populations with lower socioeconomic status may not be able to easily access optometrist services. As the differences in income account for only 20% of the impact of higher education on health behavior (50), it is clear that education is an important social determinant of health. In contrast, education level was not significantly associated with the presence of low vision or blindness after adjusting for age and gender in the study performed in Xishuangbanna Autonomous Prefecture (23). One of the most probable explanations for this is that almost 98.6% of participants had primary school-level education or below and had limited knowledge of eye healthcare. This highlighted the need to increase public awareness about eye health, especially among the poorly educated population. Educational efforts aimed at this population might increase the uptake of vision services.

## Main causes for VI

### Age-related cataract

According to the WHO, cataract, one of the most common age-related eye diseases, is the main global cause of avoidable blindness. In low- and middle-income countries, age-related cataract is responsible for 50% of blindness (51). To date, surgery is still the only way to treat cataract. Population-based studies in different regions and populations have identified significant differences in the prevalence of cataract, associated factors, and previous surgical uptake as well as visual outcomes of cataract surgery.

#### Prevalence

Age-related cataract can be classified into three main sub-types, including cortical, nuclear and posterior subcapsular cataract (PSC). The main population-based cross-sectional studies on cataract in mainland China are shown in Table 1. The overall prevalence of any cataract ranged from 20.8 to 53.1%. Of these, the prevalence of cortical cataract (10.3–31.1%) was slightly higher than that of nuclear cataract (5.1–26.1%) and PSC (1.5–4.6%) (52–54). However, in the Beijing Eye Study, nuclear cataract (grade > 3, 50.3%) was found more commonly than the cortical cataract (10.3%) (55). These discrepancies might be due to differences between the cataract grading systems used and the sampled populations. Specifically, in the Beijing Eye Study, the diagnosis of cataract was assessed using the grading system of the Age-Related Eye Disease Study (AREDS) based on slit lamp analysis and digital photographs (55); in contrast, the Handan and Wuxi studies used the LOCS III system based on slit-lamp evaluation (53, 54). To the best of our knowledge, it is difficult to make a detailed and direct comparison between these studies due to different study designs such as the different grading systems used for lens opacities and examination techniques. Therefore, there is an urgent need for researchers to conduct a nationwide population-based study to evaluate the epidemic status of cataract in China.

**Table 1:** Detailed characteristics of the main studies on cataract prevalence in mainland China.

**References**	**Survey year**	**Location**	**Setting**	**Population**	**Male (%)**	**Response rate (%)**	**Age ranges**	**Prevalence (%)**	**Male (%)**	**Female (%)**	**Definition**	**CSR**
Xu et al. (55)	2001	Beijing	Urban+ rural	4,378	43.9	84.3	≥40	53.1			Any cataract	1.3
								82			Nuclear cataract (Grade >2)	
								50.3			Nuclear cataract (Grade >3)	
								10.3			Cortical cataract	
								4.3			Posterior subcapsular cataract	4.2
Duan et al. (53)	2006–2007	Handan (Hebei)	Rural	6,830	46.3	90.4	≥30				LOCS III system grade was ≥ 2 in either eye	0.8
								20.8	17.6	23.6	Any cataract	
								5.1	4.4	5.7	Nuclear	
								18.3	14.7	21.4	Cortical	
								1.5	1.3	1.7	posterior subcapsular cataract	
Li et al. (56)	2008–2009	Mingshui (Heilongjiang)	Rural	10,384	Na	88.1	≥1	2.4			Cataract BL	Na
								3.6			Cataract UL	
Shen et al. (57)	2010	Yunnan Minority	Rural	6,546	39.8	77.8–82	≥50	7.9	6.9	8.6	Cataract social burden	6
								3.7	3.3	4	Cataract severe visual impairment or blindness	
								4.2	3.6	4.6	Presumed severe visual impairment or blindness	
Tang et al. (52)	2012	Taizhou (Jiangsu)	Rural	10,234	40.6	78.1	≥45				LOCS III system grade was ≥ 2 in either eye	
								41.6 (38.1)	40.6	42.3	Any cataract	1.45
								26.1 (24.3)	26.2	26	Nuclear	
								31.1 (28.6)	29.1	32.5	Cortical	
								4.6 (4.4)	4.4	4.7	posterior subcapsular cataract	
Jiachu et al. (58)	2017	Kandze (Sichuan)	Rural	4,764	43.8	95.3	≥50	0.61	0.34	0.82	Cataract BL	Na
								0.86	0.48	1.16	Cataract SVI	
								2.39	1.58	3.03	Cataract MVI	
								5.21	3.79	6.31	Cataract EVI	
Chen et al. (54)	2017–2018	Wuxi (Jiangsu)	Urban	6,150	45.9	91.44	≥50	23.1	20.4	25.4	Any cataract	Na
								4.9	4.6	5.2	Nuclear	
								16.4	14.1	18.3	Cortical	
								1.8	1.7	1.9	Posterior subcapsular cataract	

CSR, Cataract surgical rate; Na, not applicable; BL, bilateral blindness; SVI, severe visual impairment; MVI, moderate visual impairment; EVI, early visual impairment.

#### Incidence

Few epidemiological surveys have studied the incidence of cataract in mainland China. Only the Beijing eye study reported the 5-year incidence of age-related cataract (16.82%) (59). The 5-year incidence of cortical cataract was 11.14% which was higher than that of nuclear cataract (5.98%) and PSC (5.47%), thus indicating a large increase in the incidence of cortical cataract in China over recent years.

#### Risk factors

Many previous population-based studies revealed that the higher prevalence rate of senile cataract was associated with demographic characteristics, lifestyle, and biological factors among the Chinese population. Advanced age, female gender, lower educational levels, increased outdoor activities, reduced outdoor eye protection, higher levels of myopia, high-density lipoprotein (HDL), low-density lipoprotein (LDL), and the intake of pickled food were independently correlated with cataract and its subtypes. Furthermore, high diastolic pressure was associated with an increased likelihood of developing cortical cataract and PSC, although the mechanism involved remain unclear (60). Using a prospective design, a 5-year follow-up observation in Beijing revealed that the incidence of any cataract was independently associated with increased age and female gender, but not associated with the area of habitation, smoking or alcohol consumption. Further analysis of cataract subtypes revealed that nuclear cataract was associated with rural areas and smoking, while cortical cataract was associated with non-smoking (59). Generally, these associated factors, identified in population-based studies provide epidemiological evidence that may help prevent cataract in the Chinese population.

#### Cataract surgical rate

In our current review, we found that ~0.8–6% of the included population had undergone cataract surgeries, meaning that almost 8,000–60,000 individuals underwent cataract surgery per million of the population in China (53, 57). The highest cataract surgical rate (CSR), defined as cataract operations per million populations per year, was reported in Yunnan Minority Eye Studies (6%) (58). Although the CSR was still lower than some developing countries, such as Brazil (6.28%) (61), Nepal (7.0%) (62), and India (17.6%) (63), CRS has increased over the past two decades (from 440 in 2005 to 1067 in 2014) (64). The increasing CSR in China may be due to several factors. First, preferential policies, financial subsidies, and technical support have been provided by the Chinese government, thus attaching importance to the prevention of blindness. Second, with the rapid economic development of China, sustained effort has been made to combat cataract-related blindness over recent decades. In addition, many global health organizations provide fundamental medical access and economic supports for the prevention and treatment of cataract.

Interestingly, studies have demonstrated a “U”-shaped curve describing the relationship between CSR and age (52). In the Taizhou Eye Study, a higher CSR was reported in the population aged 45–49 years than those aged 50–69 years. Furthermore, the prevalence of cataract surgery increased among people aged 70 years or older because cataract developed significantly and severely affected the visual acuity of this group of individuals. Therefore, the health care workers should conduct more effective programs such as convenient access to medical care to increase the popularity and availability of cataract surgery in adults aged 50–69 years.

#### Cataract surgery barriers

There are several barriers that prevent cataract surgery. For example, the majority of the population did not know they had a cataract; this represents an overwhelmingly barrier for surgery. In addition, the lack of financial support for surgery, the perceived unavailability of service due to old age, a lack of knowledge regarding disease and surgery, unilateral cataracts, and the fear of surgery were also found to be barriers for surgery in China (56). These barriers could be improved by an enhanced early detection project and public health education in the future. In view of the visual needs of the growing elderly population in China, access to surgery and its related health care service must be provided, especially for those who live in rural areas. Further studies are needed to identify the most effective measure with which to promote the uptake of cataract surgery.

### Glaucoma

Glaucoma, as another main global cause of irreversible blindness, can be classified into two major types: primary open angle glaucoma (POAG) and primary angle closure glaucoma (PACG) (65). The prevalence of PACG was found to be highest in East Asians (66), while PACG affects Asians disproportionately (67). China is the world's most populous country and may have the greatest number of people who are at risk of POAG.

#### Prevalence

From 2001 to 2011, the prevalence of glaucoma ranged from 2.2 to 3.8% (Table 2). Specifically, the prevalence of POAG ranged from 0.71 to 2.85%, while the prevalence of PACG ranged from 0.7 to 1.74%. One of the reasons for the discrepancies between these studies may be differences in the examination technique and the definitions of glaucoma. In the current review, the ratio of POAG to PACG was 2.6:1 in the Beijing Eye Study (68), 1.4:1 in the Liwan Eye Study (69), and 3:1 in the Yunnan Minority Eye Study (70), thus indicating a higher prevalence for POAG than PACG. However, in the Kailu Eye Study, the proportions of POAG and PACG were almost equal (71). One of the main reasons for these discrepancies may be the age differences of participants. POAG was associated with being older (72). The participants in the Kailu study were aged 40–87 years and were younger than those who attended Beijing Eye Study (40–101 years) (68), Liwan Eye Study (50–93 years) (69), and Yunnan Minority Eye Study (50+ years) (70). Variations in environment, lifestyle, and ethnic background between the provinces of China may also be responsible for these discrepancies in prevalence.

**Table 2:** Detailed characteristics of the main studies on glaucoma prevalence in mainland China.

**References**	**Location**	**Survey year**	**Setting**	**Population**	**Male (%)**	**Response rate (%)**	**Age ranges (years)**	**Anterior chamber angle depth**	**IOP measurement**	**Optic disc evaluation**	**Definition**	**Any glaucoma (%)**	**POAG (%)**	**PACG (%)**
Wang et al. (68)	Beijing	2001	Urban+ Rural	4,315	43.8	97.2	40–101	Yes	Yes	Yes	ISGEO	3.7	2.6	1
He et al. (69)	Liwan (Guangdong)	2003	Urban	1,504	43.6	75.3	50–93	Yes	Yes	Yes, suspects	ISGEO	3.8	2.1	1.5
Liang et al. (73)	Handan (Hebei)	2007	Rural	6,716	46.4	95.5	30+	Yes	Yes	Yes	ISGEO	Na	2.2	Na
Liang et al. (74)	Handan (Hebei)	2007	Rural	6,716	46.4	95.5	30+	Yes	Yes	Yes, suspects	ISGEO	Na	Na	0.8
Qu et al. (75)	Bin (Heilongjiang)	2007	Rural	4,956	45	86.01	40+	Yes	Yes	Yes, suspects	ISGEO	Na	Na	1.57
Sun et al. (76)	Bin (Heilongjiang)	2007	Rural	4,956	45	86.01	40+	Yes	Yes	Yes, suspects	ISGEO	Na	0.71	Na
Song et al. (71)	Kailu (Inner Mongolia)	2009	Rural	5,158	44.6	87.36	40–87	Yes	Yes	Yes, suspects	ISGEO	3.28	1.42	1.74
Zhong et al. (77)	Dali (Yunnan)	2010	Rural	2,133	36.1	77.8	50+	Yes	Yes	Yes, suspects	ISGEO	2.2	Na	Na
Pan et al. (70)	Yunnan	2010	Rural	6,546	39.8	82	50+	Yes	Yes	Yes, suspects	ISGEO	3.2	2.1	0.7
He et al. (78)	Shanghai	2011	Urban	2,528	42.25	80.36	50–106	Yes	Yes	Yes, suspects	ISGEO	Na	2.85	Na

ISGEO, International Society of Geographical and Epidemiological Ophthalmology; WARMGS, Wisconsin age–related maculopathy system; PACG, primary angle-closure glaucoma; POAG, primary open-angle glaucoma; Na, not applicable.

#### Incidence

Pan et al. conducted a population-based cohort study on Bai Chinese and reported the 5-year cumulative incidence of POAG in China (79). According to their findings, ~1.3% of individuals aged 55–95 years developed POAG within 5 years. The mean annual incidence of POAG (0.26%) in the Yunnan study was lower than that in the 4-year Los Angeles study (0.78%) (80) and in the 5-year Rotterdam Eye Study (0.6%) (81), but higher than that in the 5-year Australia study (0.14%) (82). It is estimated that ~0.9 million of individuals above 50 years will develop POAG annually based on the incidence rate in the national population of China. Given the limited estimates on the incidence of POAG, there is a need to conduct a population-based and longitudinal cohort study to provide information on the prevention and control of POAG in mainland China.

#### Associated factors

Based on cross-sectional population-based evidence, the prevalence of overall glaucoma, POAG and PACG increased with age (68, 73, 75, 76, 78). However, there was no significant association between age and any form of glaucoma in Yunnan (70). In prospective studies performed in the same area, the incidences of POAG in Bai individuals aged 50–59, 60–69, and 70 years or older were 0.4, 0.6, and 2.1%, respectively, thus reflecting the fact that the incidence of POAG increased with age (79).

In addition to age, previous cross-sectional evidence indicated that family histories of glaucoma, myopia, and hypertension were also significantly associated with POAG, while PACG was associated with a family history of PACG and constipation (75). The incidence of POAG was also associated with a number of baseline variables, including advanced age, lower educational level, and the presence of myopia (79). In the Beijing Eye Study, all forms of glaucoma were correlated with age and myopia, although an association between all forms of glaucoma and male gender, Yi ethnicity, as well as myopia was observed in the Yunnan Minority Eye Study (70). In contrast, the univariable meta-regression from meta-analysis indicated that female gender was significantly associated with both POAG and PACG (11). Besides known factors, differences in social-economic factors, lifestyle, healthcare systems, and health policies between different areas could also contribute to the disparities in disease prevalence in China. A previous study indicated that individuals with dark skin color have a three-fold higher risk of POAG when compared to those with a lighter skin color (83); such individuals also develop this disease at an earlier age. Research has shown that the prevalence of POAG increases with age in Caucasian population up to 10% at the age of 90 years (83). Except for the traditional risk factors described above, pseudo-exfoliation is also known to increase the risk of POAG by a factor of 4–6-fold according to three studies in Caucasians (84–86). Young myopic men in particular (mean age 42 years) are at an increased risk of developing pigment dispersion glaucoma (87).

Generally, the increasing prevalence of both the elderly population and the comorbidities associated with glaucoma may increase significantly in the near future. Information and prevention campaigns to raise glaucoma awareness in high-risk populations and improve the available medical care may have a positive impact on visual health and need to be implemented in mainland China.

### Diabetic retinopathy

Population-based studies focusing on the epidemical evidence of diabetic retinopathy (DR) have revealed that DR is one of the major causes of VI. This condition remains a significant public health issue and the leading cause of blindness in adults of working aged (88). In China, the number of diabetic individuals is estimated to increase from 20.8 million in 2000 to 42.3 million by 2030 (89). DR is imposing a noteworthy burden on individuals, households, communities and societies (90). Currently, DR can be classified into five stages including no apparent retinopathy, mild non-proliferative retinopathy (NPDR), moderate NPDR, severe NPDR and proliferative retinopathy (PDR) (91). The Early Treatment of Diabetic Retinopathy Study (ETDRS) introduced the term clinically significant macular edema (CSME). Vision-threatening diabetic retinopathy (VTDR) is defined as the presence of severe NPDR, PDR and/or CSME (92).

#### Prevalence

In mainland China, the prevalence of DR ranged from 8.19 to 43.1% (depending on the population assessed; see Table 3). The prevalence of overall DR was reported to be 37.1% in 2001 among the subjects with a self-reported diagnosis of diabetes. Furthermore, a series of large and well-conducted population surveys over the last few years has documented a dramatic decrease in the prevalence of DR to 8.19–17.8%. Notably, there has been a gradual increase in the prevalence of DR among diabetic population-based surveys, from 24.7% in 2009 (93) to 40% in 2019 (94). However, it is important to highlight that based on diabetic population, such as those included in Table 3 (compared with general population-based designs), the assessment of DR frequency has several limitations, including potential selection bias with regards to participants. The prevalence of VTDR, PDR and CSME ranged from 2.5–4.4%, 0.9–3.3%, and 0.9–4%, respectively. One multicenter study in mainland China (including both hospital and community-based participants from 6 different provinces) reported an overall DR prevalence of 34.1% (95). Interestingly, the prevalence of DR in China varied among different provinces, with a higher prevalence in the northern regions (Beijing and Handan) than southern regions (Yangxi and Dongguan); these findings were consistent with previous meta-analyses (96, 97). Furthermore, there is no data reporting the prevalence of DR in China at the national level, and most published studies were based on regional data.

**Table 3:** Detailed characteristics of the main studies on DR prevalence in mainland China.

**References**	**Survey year**	**Location**	**Setting**	**General** **population**	**Diabetic** **population**	**Male (%)**	**Response rate (%)**	**Age ranges**	**Grading system**	**Any DR [** ***n*** **, (%)]**			**CSME** **[*n*, (%)]**	**VTDR** **[*n*, (%)]**
										**Prevalence**	**Male**	**Female**	**Prevalence**	**Prevalence**
Xie et al. (98)	2001	Beijing	Urban + Rural	4,127	232	43.5	93	40+	ETDRS	86 (37.1)	Na	Na	6 (2.6)	12 (5.2)
Xie et al. (99)	2006	Beijing	Urban + Rural	3,251	362	39.1	73.2	45–89	ETDRS	101 (27.9)	Na	Na	4 (4)	20 (5.5)
Hu et al. (100)	2006	Shenyang, Panjin, Zhuanghe, Kangping (Liaoning)	Urban + Rural	3,173	329	48.1	94.8	22–84	ETDRS	39 (11.8)	19 (5.8)	20 (6.1)	Na	Na
Wang et al. (101)	2007	Handan (Hebei)	Rural	5597	368	44.4	81.9	30+	ETDRS	165 (43.1)	60 (44.4)	105 (45.1)	13 (3.5)	23 (6.3)
Xu et al. (93)	2009	Beijing	Urban	Na	2,007	40.3	76	32–80	ETDRS	496 (24.7)	213 (10.6)	283 (14.1)	Na	Na
Yuan et al. (102)	2010–2011	Beijing	Na	2,551	614	48.2	68.9	18–79	Na	61 (9.9)	Na	Na	Na	Na
Wang et al. (103)	2011	Dalian (Liaoning)	Urban	8,391	1,809	25.4	81.5	40–90	ICDRDSS	233 (12.9)	Na	Na	Na	Na
Yang et al. (104)	2009–2012	Beijing	Urban	Na	1,340	39.78	93.5	64.8 ± 8.23	ETDRS	472 (35.22)	215 (16)	257 (19.2)	Na	Na
Cui et al. (105)	2011–2012	Dongguan (Guangdong)	Rural	8,952	1,310	40.9	87.3	46–62	ICDRDSS	233 (17.8)	125 (23)	108 (14.1)	12 (0.9)	33 (2.5)
Jin et al. (106)	2014	Yangxi (Guangdong)	Rural	5,825	476	37.2	90.7	50+	UK NDESP	39 (8.19)	(6.21)	(9.36)	Na	Na
Pan et al. (107)	2015	Suzhou (Jiangsu)	Urban	Na	880	44	96.4	67.7 ± 8.3	ETDRS	158 (18)	78 (20.2)	80 (16.3)	Na	39 (4.4)
Yin et al. (94)	2018–2019	Shijiazhuang (Hebei)	Urban	Na	1,008	46	98.6	58.13 ± 8.26	IRIS	409 (40)	219 (21.7)	380 (37.6)	Na	Na

DR, Diabetic Retinopathy; ICDRDSS, International Clinical Diabetic Retinopathy Disease Severity Scale; IRIS, Intelligent Retinal Imaging System; UK NDESP, UK National Diabetic Eye Screening Programme; ETDRS, Early Treatment Diabetic Retinopathy Study; Na, not applicable.

#### Incidence

Currently, there are few large-scale population-based studies on the incidence of DR in mainland China (108, 109). Between 2001 and 2011, the cumulative 10-year incidence of DR among general participants without DR at baseline aged 40 years or older was 4.2% per year, with a mean annual incidence rate of being ~0.42% during this period (109). In the same study in Beijing, the 5-year (2001–2006) progression rate of DR was 21% and the annual progression rate was 4.2% when considering existing DR in individuals with known diabetes (108). Collectively, these findings suggested a considerable burden of DR in mainland China.

#### Associated factors

Except for common factors associated with the presence of DR, such as living in rural regions, the duration of diabetes, the use of anti-diabetes medications, blood pressure, fasting plasma glucose and glycated hemoglobin A_1_c (HbA_1_c) levels, the target factors reported in other large studies from China included age, gender, cataract surgery and refractive error (101, 105–107). In a study by Pan et al., advanced age was found to be associated with a lower risk of DR but a higher risk of VTDR. Unsurprisingly then, the association between younger age and the risk of DR has been reported in many other studies, especially in China (93) and India (110). This might be explained by the importance of dietary patterns in terms of a younger lifestyle, including the consumption of more meat products or drinking more alcohol, thus leading to an increased risk of DR. Designers of future studies relating to DR may consider including the dietary patterns or other parameters associated with the lifestyle background in study designs. In the Yangxi study, previously undergone cataract surgery may increase the risk of DR (106), which is consistent with previous studies (108, 111, 112). Surprisingly, cataract surgery with complications could increase the post-operative risks for NPDR, and this influence might persist for five years after surgery (112). Furthermore, in addition to the factors summarized above, the Beijing Eye study (2006) reported a marginally significant association between DR and hyperopia (99). This might be explained from the perspective of the correlation between myopia and DR. Previous meta-analyses suggested that individuals with myopia were associated with a reduced risk of developing DR or VTDR (113), and a longer axial length protected against DR (114).

In the population-based longitudinal Beijing Eye Study, the 10-year incidence of DR was significantly associated with higher HbA_1_c levels, a longer duration of diabetes, higher serum concentrations of creatinine, lower educational levels, higher estimated cerebrospinal fluid pressure and shorter axial length (109). Furthermore, the 5-year progression of DR was associated with rural regions and self-reported arterial hypertension (108). Considering the limited reports available, the insights provided by further longitudinal research in DR may help to improve the effectiveness of ongoing public health policies and to help to develop DR prevention and control interventions.

### Age-related macular degeneration

Age-related macular degeneration (AMD) is one of the most common age-related eye diseases resulting in VI or even blindness worldwide (115). At present, the number of individuals with AMD is experiencing a gradual increase in mainland China with the aging process. It is estimated that from 2020 to 2050, the number of cases of any form of AMD will rise by 76.72% in China, from 31.23 to 55.19 million (10). However, substantial epidemiological data on the global effects of AMD are limited. There are two common grading systems for AMD classification in epidemic research, including the Wisconsin Age-Related Maculopathy Grading System (116) and the Beckman Initiative for Macular Research Classification Committee (117). AMD is classified into early, intermediate and late stages. Accurately determining the epidemiology of AMD is important in order to develop preventive measures for this disease.

#### Prevalence

Five population-based surveys have been carried out to investigate the epidemiological features of AMD in mainland China (Table 4). From 2000 to 2015, the all-age number and rate for AMD prevalence increased significantly in China, which could largely be due to the increasing and aging population. This temporal trend in the prevalence of AMD in China was most similar to that of findings from the GBD 2019 (118). Notably, the age standardized disability-adjusted life years (DALYs) rate of AMD has exhibited a slight decrease over recent years in China with the improvement of diagnostic tools and treatments, whereas the burden of AMD decreased in individuals aged 60–85 years; however, this disease burden is still much higher when compared to neighboring developed countries (118). Therefore, a more thorough screening strategy should be utilized for residents aged 60–85 years. Early AMD mostly occurred in individuals who were 50–59 years-of-age and 60–69 years-of-age while intermediate or late AMD mostly occurred in those who were 70–79 years-of-age and older than 80 years-of-age, respectively (119). Total AMD was more prevalent in males than in females (62.8 vs. 57.1%) (120). The existence of gender differences in AMD that are mediated by gender for AMD may be largely attributed to substantially higher proportions of smokers in Chinese men than in women (121). From a national perspective, insufficient data are available for the national estimates and projections of AMD.

**Table 4:** Detailed characteristics of the main studies on AMD prevalence in mainland China.

**References**	**Survey year**	**Location**	**Setting**	**General population**	**Male (%)**	**Response rate (%)**	**Age ranges**	**Grading system**	**Overall [** ***n*** **, (%)]**				**Male [** ***n*** **, (%)]**				**Female [** ***n*** **, (%)]**			
									**Any AMD**	**Early**	**Intermediate**	**Late**	**Any AMD**	**Early**	**Intermediate**	**Late**	**Any AMD**	**Early**	**Intermediate**	**Late**
Li et al. (122)	2001	Beijing	Urban+Rural	4376	Na	83.4	≥40	W	70 (1.6)	63 (1.4)	Na	7 (0.2)	Na	Na	Na	Na	Na	Na	Na	Na
Yang et al. (121)	2006- 2007	Handan (Hebei)	Rural	6,581	46.5	96.4	≥30	W	204 (3.1)	200 (3.1)	Na	4 (0.1)	122 (4)	119 (3.9)	Na	3 (0.1)	82 (2.33)	81 (2.3)	Na	1 (0.03)
Ye et al. (123)	2012- 2013	Jiangning (Shanghai)	Urban	2,005	43.7	82.5	≥50	W	229 (11.4)	206 (10.3)	Na	23 (1.1)	116 (5.8)	100 (11.4)	Na	16 (1.8)	113 (5.6)	106 (9.4)	Na	7 (0.6)
Jin et al. (120)	2014	Yangxi (Guangdong)	Rural	4,881	49.1	83.8	≥50	B	2,924 (59.9)	2,003 (41.0)	879 (18.0)	42 (0.9)	1,505 (62.8)	982 (41.0)	495 (20.6)	28 (1.2)	1,419 (57.1)	1,021 (41.1)	384 (15.5)	14 (0.6)
Zhang et al. (119)	2015	Haikou, Sanya, Wanning and Dongfang (Hainan)	Urban+Rural	2,232	34.4	95.3	≥50	B	357 (15.6)	267 (11.7)	64 (2.8)	24 (1.1)	141 (18.5)	Na	Na	Na	206 (14.7)	Na	Na	Na

AMD, age-related macular degeneration; W, Wisconsin Age-Related Maculopathy Grading System; B, Beckman Initiative for Macular Research Classification Committee; Na, not applicable.

Optical coherence tomography (OCT) facilitates *in vivo* evaluation of the retina at a near-cellular level, and has increasingly become a vital tool in clinical practice for the diagnosis and management of AMD. Although OCT is widely available in China, only the Jiangning Eye study utilized OCT (123); most studies focused more on fundus photography. Moreover, in population or community-based studies, fluorescein angiography (FFA) and indocyanine green angiographies (ICGA) cannot be performed routinely. Furthermore, it is difficult to diagnose the polypoidal choroidal vasculopathy (PCV) from fundus photographs alone. Therefore, the prevalence of AMD, particularly late AMD, may be overestimated. However, owing to the limited number of subjects with late AMD, this weakness would not have had a marked influenced on previous findings.

#### Incidence

Few cohort studies have focused on the incidence of AMD among the population living in mainland China (124, 125). In both Beijing and Handan eye studies, the incidence of early and late AMD over a 5-year period was ~4.2% and 0.1–0.24%, respectively. The incidence of early and late AMD was markedly less prevalent in China than that in the Beaver Dam Eye Study (early/late AMD, 8.19%/0.91%) (126), the Blue Mountain Eye Study (early/late AMD, 8.74%/1.10%) (127), the Hisayama Study (early/late AMD, 7.95%/0.84%) (128), and the Singapore Malay Eye Study (early/late AMD, 5.09%/0.72%) (129). Globally, the pooled annual incidence rates of early and late AMD were 1.59 and 0.23 per 100 person-years in our meta-analysis, respectively (115), which was higher than that in mainland China (early/late AMD, ~0.8%/0.04%) (124, 125). Most of the global reports are based on data from developed countries. It is foreseeable that the incidence of AMD may become more severe with the continuous development of China's economy, the aggravation of air pollution and the extension of life expectancy.

The Beaver Dam Eye Study has completed a 20-year observation of the incidence of AMD in 2014 (130); in contrast, reports from mainland China only reported data over a 5-year period. Further large-scale longitudinal epidemiological studies are also required to better develop eye-care strategies and health services, especially with regards to AMD.

#### Associated factors

As an age-related degeneration disease, age is considered as the most important risk factor for AMD (119, 123). Other associated factors included educational level, smoking, outdoor activities and diet for any forms of AMD, whereas axial myopia was shown to be negatively associated with early AMD (119, 123). Research also showed that age, male gender, and an increased in axial length were significantly associated with the incidence of early AMD in Handan (125). In addition, a more advanced age at baseline, a smaller optic disc size, a smaller scleral spur distance, and hyperopic refractive error were all associated with the occurrence of early AMD in Beijing (124). Globally, smoking was shown to be an independent risk factor for both early and late AMD, whereas age, HDL, and alcohol consumption were factors associated with the incidence of early AMD (115).

## Perspectives on prevention

### Visual impairments

In 2019, the age-standardized prevalence of vision loss was 2.57% for moderate vision impairment, 0.25% for severe vision impairment and 0.48% for blindness in China. These rates were all below the global average, although the prevalence of moderate and severe vision impairment increased more rapidly than in other Group of 20 (G20) countries from 1990 to 2019 (3). Globally, the crude all-age prevalence of individuals who were blind and had a PVA of worse than 3/60 in the better eye was 0.55%, which is likely to be higher than that in China (131). Similarly, the prevalence of VI increased with age and the main causes of VI varied across age groups in both China and the rest of the world (3, 131). The leading causes of VI in China were uncorrected refractive error, cataract and AMD in both 1990 and 2019 in the overall population (32). Globally, the leading causes of blindness were cataract, uncorrected refractive error, glaucoma, AMD and DR (132). This discrepancy should be noted as a significant gap for eye care in China. Under-corrected or uncorrected refractive error was the leading cause of presenting visual impairment (PVI) and even blindness (133). The estimated half of individuals with PVI could be improved by the effects of wearing better-correcting glasses (36). However, the unawareness of the need for correcting spectacles and the cost of spectacles use still may present barriers to some patients. To date, the medical costs of refractive error correction have not been covered by the Urban-Rural Resident Basic Medical Insurance Scheme (URRBMI). Herein, more attention on screening, education, and financial support of refractive error to encourage adequate spectacle coverage could have important public health implications in China (134).

### Cataract

Age-related cataract is the leading cause of remediable blindness among the elderly population worldwide and surgery is the most common first-line procedure performed in therapeutic approaches for cataract (135). Although Chinese governments have promoted the consolidation and re-distribution of health care services over past few decades, the uneven distribution of health care resources is still apparent, especially in rural regions (136). The CSR in urban areas was relatively higher than that in rural regions (53, 54), and most experienced cataract surgeons in China were concentrated in urban hospitals. Therefore, strengthening the availability of eye care and providing sufficient training opportunities for junior ophthalmologists in rural and remote regions would be the most effective way with which to reduce cataract blindness in China.

### Glaucoma

Glaucoma is the leading cause of irreversible blindness worldwide, thus, early detection and treatment are crucial ways for preventing VI resulting from this relatively asymptomatic initial phase of the disease. The diagnosis of glaucoma requires comprehensive consideration of multiple indicators, including the visual field, the angle width of the anterior chamber and changes in the optic disc and retina by experienced ophthalmologists (137). However, the extremely limited glaucoma specialists, general ophthalmologists, ophthalmic graders, eye clinics or hospitals pose a significant problem in China; this may lead to a high rate of misdiagnosis and delayed treatment (138). Therefore, promoting public screening and health education regarding glaucoma will be effective for disease control in China. A study using a decision-analytic Markov model revealed that combined screening for POAG and PACG in China is likely to be cost-effective, apparently due to the relatively low costs of screening, particularly labor costs, and the high risk of blindness in untreated cases, particularly those with PACG (139).

### Diabetic retinopathy

Recently, retinopathy has risen to be the main cause of VI in the elderly population. In order to implement the “Thirteenth Five-Year Plan for National Eye Health (2016–2020)” (140), and to address the sustainability of DR management, Chinese governments have formulated the “Technical Plan for Diabetic Retinopathy Graded Diagnosis and Treatment Service,” hoping to achieve early detection and treatment of DR, and reduce the burden of disease.

A three-level (provincial, municipal and county levels) DR prevention network platform has been promoted in some regions of China. Primary prevention, located in county or community health centers, focuses on preventing or delaying the onset of DR in residents with diabetes by lifestyle interventions (e.g., diet and exercise), anti-diabetes and anti-hypertension medications, and regular screening to detect the early onset of DR. Secondary prevention, located in municipal or second class hospitals, focused on controlling the progression of DR by addressing the control of associated risk factors, regular screening to detect the early onset of any VTDR stages and the referral of DR patients in need of treatment to higher-level medical institutions in a timely manner according to specific guidelines (141). In this aspect, remote medical consultation or artificial intelligence aided diagnosis tools offers promising platforms for the monitoring of DR, particularly in underdeveloped regions. Third prevention strategies, located in provincial or tertiary hospitals, focus on the treatments, such as laser, anti-vascular endothelial growth factor (anti-VEGF) therapy, steroids, and vitrectomy for VTDR. Generally speaking, to tackle DR in mainland China, it is necessary to consider a collaborative approach between the patient, the primary care physician, and subspecialists in the management of the patient's systemic disorder, with specific attention to the control of blood sugar, blood pressure, serum lipids, body weight, and the lifelong monitoring of retinopathy progression.

### Age-related macular degeneration

With an aging population and falling death rates in mainland China, the prevalence and disease burden of AMD is likely to become a greater public health concern in the coming decades. Thus, to prevent the disease, it is important to identify early of modifiable risk factors early, such as diet and lifestyle (21). Critically, current epidemiological information (e.g., prevalence, incidence and associated factors) offers detailed insights into the public health burden of AMD in mainland China. Herein, these findings can serve as a reference for health policy decision-making frameworks and resource allocation in AMD control and prevention strategies. A further point to raise is that the number of late AMD cases in China rose from 2.58 to 5.74 million between 1990 and 2005. In addition, neovascular AMD (nvAMD) is anticipated to exhibit an increased rate of 57.48%, from 0.78% in 2020 to 1.22% in 2050, whereas the increasing rate of early AMD will be the smallest (at 38.45%) from 4.23 to 5.21% during the same period. In view of the large population size in China, this striking finding highlights an urgent need for action with regards to the prevention and treatment of late AMD, given its clinical significance (10). With regards to secondary prevention, more attention should be paid to the health care costs of late AMD, especially for nvAMD for which progression to the loss of sight could be slowed by photodynamic therapy (PDT) or intravitreal (IVT) anti-VEGF. Anti-VEGF therapies are considered as first-line treatments for nvAMD in China. However, the expensive costs and frequent follow-up or injections associated with this treatment may inhibit patients in mainland China from seeking medical care. Consequently, the optimal allocation of resources in healthcare could ensure that those with a high risk for AMD undergo regular eye cares appointments and receive timely interventions; this may be an effective public health strategy. Annual screening for AMD based on a teleophthalmology platform has been proven to be an optimal screening approach (142).

## Current challenges and the way forward

However, multiple challenges and obstacles still need to be overcome, including the accelerated aging of the Chinese population, the lack of structured care delivery in many underdeveloped regions, and unequal access to appropriate care.

Optimal age-related avoidable blindness (e.g., uncorrected refractive error and cataract) management requires not only fundamental medical access, but also economic factors. In addition, the main causes of irreversible blindness (e.g., glaucoma, DR and AMD) require patient empowerment, health literacy, self-management and self-discipline (143). Chinese populations living in remote areas continue to regard poor vision as a natural process of aging and are lacking public awareness and knowledge on eye diseases. However, there is a relative lack of eye health educators in China, and support provided by tele-consultation may also be helpful (144).

In the “Thirteenth Five-Year Plan for National Eye Health (2016–2020),” Government and health policy makers proposed development of the Chinese eye health management system to address the sustainability of eye health management and the feasibility of payment for eye health (140). At present, these organizations are formulating the “Fourteenth Five-Year Plan for National Eye Health” with the aim of meeting the eye health needs of the Chinese population.

While many studies have improved our understanding of the epidemiology of VI and age-related eye diseases in mainland China, many gaps in knowledge still remain. The following needs have been identified:

i. Ongoing research covering national surveys measuring the presence and incidence of common age-related eye diseases such as refractive error, cataract, glaucoma, DR and AMD, and those age-related eye diseases not routinely measured in population-based epidemiologic studies including ischemic optic neuropathy and less common retinal conditions;
ii. Well-designed population-based eye studies without demographic differences in terms of age and gender should be conducted, which may provide an exact estimation of the epidemic information;
iii. Improved knowledge of age-related eye diseases based on new guidelines or grading systems to assess each condition and establish a consensus on how to prevent these conditions;
iv. Validate new technologies, such as optical coherence tomography angiography (OCTA) should be used to detect manifestations or biomarkers in patients with age-related eye diseases (e.g., AMD) along with additional objective measurements for grading and management;
v. Identify cost-effective health economics and quality-of-life analysis in epidemiological cross-sectional or cohort studies;
vi. Educate the public and Congress on how to integrate epidemiological research findings into useful applications into basic sciences, behavioral and clinical practices, for better public health according to social-economic development.

To summarize, although the Chinese Fourteenth Five-Year Plan to prevent blindness is in place, public awareness of eye health needs to be improved, and preventing predominant eye diseases and reducing the prevalence of VI should be prioritized in future research.

## Author contributions

LL: conception or design of the work. CL, BZ, and LL: drafting the article. XY, HY, and LL: critical revision of the manuscript. All authors: final approval of the manuscript.

## Funding

This work was supported by GDPH Supporting Fund for Talent Program (KY0120220263), Science and Technology Program of Guangzhou, China (202002020049 and 20220610092), Project of Special Research on Cardiovascular Diseases (2020XXG007), National Medical Simulation Education Research Project (2021MNYB01), National Natural Science Foundation of China (82271125, 81870663, and 82171075), and the Outstanding Young Talent Trainee Program of Guangdong Provincial People's Hospital (KJ012019087).

## Conflict of interest

The authors declare that the research was conducted in the absence of any commercial or financial relationships that could be construed as a potential conflict of interest.

## Publisher's note

All claims expressed in this article are solely those of the authors and do not necessarily represent those of their affiliated organizations, or those of the publisher, the editors and the reviewers. Any product that may be evaluated in this article, or claim that may be made by its manufacturer, is not guaranteed or endorsed by the publisher.
