# Supplementary material for: Epidemiology, health policy and public health implications of visual impairment and age-related eye diseases in mainland China
Source: Front Public Health. 2022 Nov 9;10:966006. doi: 10.3389/fpubh.2022.966006 (PMC9682104; doi:10.3389/fpubh.2022.966006)
Supplement: Supplementary file 1 [file Table_1.DOCX]

**Supplementary Table S1. Detailed characteristics of the main studies on visual impairment prevalence in mainland China.**

| **Study** | **Survey year** | **Location** | **Setting** | **Population** | **Male (%)** | | **Response rate (%)** | | **Age ranges** | | **Instrument and measurement** | | **Criteria** | **Definition** | **Prevalence (%)**  **Total/Male/Female** | | | **Main cause** | **Risk factors** |
| --- | --- | --- | --- | --- | --- | --- | --- | --- | --- | --- | --- | --- | --- | --- | --- | --- | --- | --- | --- |
|  |  |  |  |  |  |  |  |  |  |  |  |  |  |  | **VI** | **Low vision** | **Blindness** |  |  |
| Zhao et al, 1998(1) | 1996 | Shunyi (Beijing) | Rural | 5084 | 44 | | 91.5 | | ≥50 | | ETDRS E-chart, PVA | | WHO | VI: less than 6/18 to 6/60 or greater in worse eye, 6/60 or greater in better eye;  Moderate bilateral blindness: less than 6/60 in worse eye, less than 6/60 to 3/60 or greater in better eye;  Severe bilateral blindness: less than 3/60 in both eyes | 18.2/ 16.0/ 20.0 |  | Moderate: 0.9/ 0.6/ 1.1;  Severe: 1.9/ 1.1/ 2.4 | VI: refractive error, cataract, retinal abnormalities;  Moderate bilateral blindness: amblyopia, retinal abnormalities, corneal scar;  Severe bilateral blindness: glaucoma, corneal scar, retinal abnormalities |  |
| Li et al, 1999(2) | 1997 | Doumen (Guangdong) | Rural | 5426 | 45.3 | | 84.2 | | ≥50 | | ETDRS E-chart, PVA | | WHO | VI: less than 0.32 to 0.10 or greater in worse eye, 0.10 or greater in better eye;  Moderate bilateral blindness: less than 0.10 in worse eye, less than 0.10 to 0.05 or greater in better eye;  Severe bilateral blindness: less than 0.05 in both eyes | Total:25.53 |  | Moderate: 1.70;  Severe: 2.67 | VI: cataract, refractive error, corneal scar | VI: age, sex, education |
|  |  |  |  |  |  | |  | |  | | ETDRS E-chart, Pinhole VA | | WHO | VI: less than 0.32 to 0.10 or greater in worse eye, 0.10 or greater in better eye;  Moderate bilateral blindness: less than 0.10 in worse eye, less than 0.10 to 0.05 or greater in better eye;  Severe bilateral blindness: less than 0.05 in both eyes | Total: 13.79 |  | Moderate: 1.10;  Severe: 2.34 |  |  |
| Xu et al, 2006(3) | 2001 | Beijing | Urban+ rural | 4409 | 43.8 | | 83.4 | | ≥40 | | Snellen charts, BCVA | | WHO | Low vision: VAs in the better-seeing eye of 20/60 to 20/400  Blindness: VAs in the better-seeing eye < 20/400 |  | 1.1 (Urban: Rural= 0.9: 1.4)/ 0.7/  1.5 | 0.3 (Urban: Rural=0.2: 0.5)/ 0.2/ 0.4 | Low vision: cataract, degenerative myopia, glaucoma;  Blindness: cataract, corneal opacity, degenerative myopia | Low vision: female, illiteracy, age, high myopia;  Blindness: age, illiteracy |
|  |  |  |  |  |  | |  | |  | | Snellen charts, BCVA | | US | Low vision: BCVA in the better-seeing eye of < 20/40 and >= 2/20  Blindness: BCVA in the better-seeing eye of < 2/20 |  | 2.2 (Urban: Rural= 1.5: 3.0)/ 1.3/  2.8 | 0.3 (Urban: Rural=0.2: 0.5)/ 0.2/ 0.4 | Low vision: cataract, degenerative myopia, glaucoma;  Blindness: cataract, degenerative myopia, corneal opacity | Low vision: rural, female, illiteracy, age, high myopia; Blindness: age, illiteracy, high myopia |
| Huang et al, 2009(4) | 2003-2004 | Liwan (Guangdong) | Urban | 1405 | 43.4 | | 75.3 | | ≥50 | | ETDRS E-chart, PVA | | WHO | VI: VA of less than 20/63 in the better-seeing eye;  Low vision: VA <20/63–20/400 in the better-seeing eye;  Blindness: VA <20/400 in the better-seeing eye | Total: 16.2 | Total: 10.1 | Total: 0.6 | VI: cataract, refractive error, glaucoma;  Low vision: cataract, refractive error, glaucoma;  Blindness: cataract, glaucoma, refractive error |  |
|  |  |  |  |  |  | |  | |  | | ETDRS E-chart, BCVA | | WHO | VI: VA of less than 20/63 in the better-seeing eye;  Low vision: VA <20/63–20/400 in the better-seeing eye;  Blindness: VA <20/400 in the better-seeing eye | Total: 6.1 | Total: 3.1 | Total: 0.5 | VI: cataract, AMD, myopic maculopathy;  Low vision: cataract, AMD, myopic maculopathy;  Blindness: cataract, glaucoma, myopic maculopathy |  |
|  |  |  |  |  |  | |  | |  | | ETDRS E-chart, PVA | | US | Low vision: VA of less than 20/40 but more than 20/200 in the better-seeing eye;  Legal blindness: VA of not more than 20/200 |  | Total: 9.5 | Total: 1.2 |  |  |
|  |  |  |  |  |  | |  | |  | | ETDRS E-chart, BCVA | | US | Low vision: VA of less than 20/40 but more than 20/200 in the better-seeing eye;  Legal blindness: VA of not more than 20/200 |  | Total: 2.9 | Total: 0.6 |  |  |
| Wu et al, 2008(5) | 2006 | Kunming (Yunnan) | Urban+ rural | 2588 | 43.3 | | 93.8 | | ≥50 | | RAAB tumbling “E” chart, PVA | | WHO | VI: PVA >= 6/60 to < 6/18 in the better eye;  SVI: PVA >= 3/60 to < 6/60 in the better eye;  Blindness: presenting VA < 3/60 in the better eye | VI: 9.1;  SVI: 3.0 |  | Total: 3.7 | VI: cataract, refractive error, other posterior diseases;  SVI: cataract, other corneal scar, other posterior diseases;  Blindness: cataract, other corneal scar, glaucoma |  |
| Zhao et al, 2010(6) | 2006 | Beijing, Jiangsu, Guangdong, Heilongjiang, Hebei, Jiangxi, Chongqing, Yunnan, Xinjiang | Rural | 45747 | 45.8 | | 90.8 | | ≥50 | | LogMAR E chart, PVA | | WHO | VI: VA <20/63 to ≥20/400 in the better eye;  Blindness: VA <20/400 in the better eye | 10.8/ 9.31/ 12.0 |  | 2.29/ 1.83/ 2.68 |  |  |
|  |  |  |  |  |  | |  | |  | | LogMAR E chart, BCVA | | WHO | VI: VA <20/63 to ≥20/400 in the better eye;  Blindness: VA <20/400 in the better eye | 5.30/ 4.47/ 6.00 |  | 1.93/ 1.53/ 2.27 |  |  |
| Li et al, 2015(7) | 2006 | Shanxi | Urban+ rural | 75016 | 50.8 | | 85.5 | |  | | LogMAR E chart, BCVA | | WHO | VI: VA<6/18 in the better eye;  Blindness: VA<3/60 in the better eye or visual field restriction to no more than the central 10° | 0.44/ 0.31/ 0.57 |  | 0.19/  0.13/ 0.24 | VI: cataract, retinopathy/ choroidopathy, hereditary and developmental disorders;  Blindness: cataract, retinopathy/ choroidopathy, hereditary and developmental disorders |  |
| Guo et al, 2017(8) | 2006 | the second China National Sample Survey Disability | Urban+ rural | 1,909,199 | 49.7 | | 75.6 | | ≥18 | | LogMAR E chart, BCVA | | WHO | VI: BCVA < 0.3;  Low vision: 0.05 ≤ BCVA < 0.3;  Blindness: no light perception ≤ BCVA < 0.05, visual field less than 10 degrees; the better-seeing eye | 1.7 (Urban: Rural=1.1:1.9)/ 1.3/ 2.0 | 1.1 (Urban: Rural= 0.8: 1.2)/ 0.9/  1.3 | 0.58 (Urban: Rural=0.3: 0.7)/ 0.4/ 0.7 | VI: cataract, disorders of choroid and retina, and disorders of cornea | VI: age, gender, rural residents |
| Liang et al, 2008(9) | 2006-2007 | Handan (Hebei) | Rural | 6830 | 90.4 | |  | | ≥30 | | LogMAR E chart, PVA | | WHO | Low vision: VA was 20/60 or worse but 20/400 or better;  Blindness: VA was worse than 20/400 in the better eye |  | 4.7/ 3.8/  5.7 | 0.6/ 0.3/ 1.0 | Low vision: undercorrected refractive error, cataract, myopic maculopathy;  Blindness: undercorrected refractive error, myopic maculopathy, cataract |  |
|  |  |  |  |  |  | |  | |  | | LogMAR E chart, BCVA | | WHO | Low vision: VA was 20/60 or worse but 20/400 or better;  Blindness: VA was worse than 20/400 in the better eye |  | 1.0/ 0.8/  1.3 | 0.5/ 0.2/ 0.8 | Low vision: cataract, myopic maculopathy, glaucoma  Blindness: cataract, myopic maculopathy, glaucoma |  |
| Song et al, 2010(10) | 2007 | Bin County (Heilongjiang) | Rural | 4956 | 44.9 | | 86.01 | | 40-89 | | ETDRS E-chart, PVA | | WHO | VI: VA <20/63 in the better eye;  Low vision: VA <20/63 to ≥20/400 in the better eye;  Blindness: VA <20/400 in the better eye | 9.6/ 7.5/ 11.3 | 7.7/ 5.6/  9.3 | 1.9/ 1.9/  2.0 | VI: refractive error, cataract, glaucoma;  Blindness: cataract, glaucoma, corneal disorders | VI: age, gender, education |
|  |  |  |  |  |  | |  | |  | | ETDRS E-chart, BCVA | | WHO | VI: VA <20/63 to ≥20/400 in the better eye;  Blindness: VA <20/400 in the better eye | 6.6/ 5.5/ 7.6 | 4.9/ 3.8/  5.8 | 1.7/ 1.7/  1.8 | VI: cataract, glaucoma, corneal disorders;  Blindness: cataract, glaucoma, corneal disorders | VI: age, gender, education |
| Xiao et al, 2010(11) | 2007 | Gao`an, Xin gan, Wan zai (Jiangxi) | Rural | Gao`an (4699), Xin gan (3834), Wan zai (2861) | Gao`an (44.9), Xin gan (44.4), Wan zai (47.3) | | Gao`an (94), Xin gan (95.9), Wan zai (95.4) | | ≥50 | | RAAB tumbling ‘E’ chart, PVA | | WHO | VI: VA <6/18 to 6/60;  SVI: VA<6/60 to 3/60;  Blindness: VA<3/60 in the better eye | VI: 6.5/ 5.0/ 7.7 (Gao`an), 5.8/ 5.2/ 6.3 (Xin gan),5.1/ 4.1/ 6.0  (Wan zai); SVI: 1.4/ 0.8/ 1.9 (Gao`an), 1.3/ 0.8/ 2.5 (Xin gan),1.3/ 0.9/ 1.6  (Wan zai) |  | 1.5/ 1.0/ 1.9 (Gao`an), 1.8/ 0.9/ 2.5 (Xin gan),1.6/ 1.9/ 1.3  (Wan zai) | VI: Gao`an (Uncorrected refractive error), Xin gan (Uncorrected refractive error), Wan zai (Uncorrected refractive error);  SVI: Gao`an (Cataract), Xin gan (posterior segment disorders), Wan zai (Cataract);  Blindness: Gao`an (Cataract), Xin gan (Cataract), Wan zai (Cataract) |  |
| Li et al, 2008(12) | Na | Harbin (Heilongjiang) | Rural | 5057 | 47.1 | | 91 | | ≥50 | | LogMAR E chart, PVA | | WHO | Bilateral VI: PVA worse than 20/60 but equal to or better than 20/400 in the better eye;  Bilateral blindness: PVA worse than 20/400 in the better eye | 8.3/ 6.0/ 10.3 |  | 1.9/ 1.1/ 2.3 | VI: cataract, refractive error, AMD;  Blindness: cataract, corneal opacity, AMD |  |
| Cheng et al, 2016(13) | 2009 | Kailu (Inner Mongolia) | Rural | 5158 | 44.6 | | 87.4 | | ≥40 | | LogMAR E chart, PVA | | WHO | Low vision: distance VA of <20/60, but equal to or better than 20/400;  Blindness: distance VA of <20/400 | Na | 7.52/ 6.83/ 8.08 | 2.23/2.13/ 2.31 | Low vision: cataract, refractive error, glaucoma;  Blindness: cataract, refractive error, glaucoma | Low vision: age, female, low income |
|  |  |  |  |  |  | |  | |  | | LogMAR E chart, BCVA | | US | Low vision: distance VA of <20/60, but equal to or better than 20/400;  Blindness: distance VA of <20/400 |  | 3.80/ 2.96/ 4.48 | 0.89/ 0.96/ 0.84 | Low vision: cataract, glaucoma, posterior segment disorders;  Blindness: cataract, glaucoma, posterior segment disorders | Low vision: age, female, low education |
| Li et al, 2012(14) | 2010 | Dali (Yunnan) | Rural | 2133 | | 36.1 | | 77.8 | | ≥50 | | ETDRS E-chart, PVA | WHO | VI: VA of less than 20/63 in the better-seeing eye;  Low vision: VA <20/63–20/400 in the better-seeing eye;  Blindness: VA <20/400 in the better-seeing eye | Total: 18.91 | 15.22/ 13.59/ 16.15 | 3.59/ 3.27/ 3.78 | VI: cataract, refractive error, myopic maculopathy;  Low vision: cataract, refractive error, AMD;  Blindness: cataract, myopic maculopathy, glaucoma |  |
|  |  |  |  |  | |  | |  | |  | | ETDRS E-chart, BCVA | WHO | VI: VA of less than 20/63 in the better-seeing eye;  Low vision: VA <20/63–20/400 in the better-seeing eye;  Blindness: VA <20/400 in the better-seeing eye | Total: 10.45 | 7.75/ 6.14/ 8.67 | 2.88/2.35/ 3.19 | VI: Cataract, AMD, myopic maculopathy;  Low vision: cataract, AMD, myopic maculopathy;  Blindness: cataract, glaucoma, corneal opacity |  |
|  |  |  |  |  | |  | |  | |  | | ETDRS E-chart, PVA | US | Low vision: VA of less than 20/40 but more than 20/200 in the better-seeing eye;  Legal blindness: VA of not more than 20/200 |  | Total: 24.82 | Total: 8.13 |  |  |
|  |  |  |  |  | |  | |  | |  | | ETDRS E-chart, BCVA | US | Low vision: VA of less than 20/40 but more than 20/200 in the better-seeing eye;  Legal blindness: VA of not more than 20/200 |  | Total: 13.48 | Total: 4.96 |  |  |
| Li et al, 2013(15) | 2010 | Haikou (Hainan) | Rural | 6482 | 46.4 | | 95.3 | | ≥45 | | RAAB A tumbling “E” chart, PVA | | WHO | VI: PVA <6/18 but ≥6/60 in the better eye;  SVI: PVA <6/60 but ≥3/60 in the better eye;  Blindness: PVA <3/60 in the better eye | VI: 8.4/ 7.1/ 9.8;  SVI: 1.6/ 1.1/ 2.0 |  | 3.5/ 2.1/ 5.0 | VI: refractive error, cataract, other posterior segment disorder;  SVI: cataract, other posterior segment disorder, refractive error;  Blindness: cataract, other posterior segment disorder, corneal scar |  |
| Zhang et al, 2017(16) | 2012 | Chaonan (Guangdong) | Rural | 3484 | 40.1 | | 94.2 | | ≥50 | | RAAB (LogMAR) tumbling E chart, PVA | | WHO | VI: VA 6/60 and <6/18;  SVI: VA 3/60 and <6/60;  Blindness: VA <3/60 | VI: 6.4/ 4.5/ 8.2;  SVI: 1.0/ 0.8/ 1.3 | Na | 2.4/ 1.5/ 3.3 | VI: refractive error, cataract, posterior segment disorders;  SVI: cataract, posterior segment disorders, refractive error;  Blindness: cataract, posterior segment disorders, refractive error |  |
| Tang et al, 2015(17) | 2012-2013 | Taizhou Zhejiang) | Rural | 10234 | 40.7 | | 78.1 | | ≥45 | | LogMAR E chart, PVA | | WHO | Low vision: BCVA <20/63 - 20/400 in the better eye;  Blindness: BCVA <20/400 in the better eye |  | 8.0/ 6.5/  8.8 | 1.0/ 0.8/ 1.2 |  |  |
|  |  |  |  |  |  | |  | |  | | LogMAR E chart, BCVA | | WHO | Low vision: BCVA <20/63 - 20/400 in the better eye;  Blindness: BCVA <20/400 in the better eye |  | 5.1/ 4.0/  5.7 | 1.0/ 0.7/ 1.2 |  |  |
|  |  |  |  |  |  | |  | |  | | LogMAR E chart, PVA | | US | Low vision: BCVA <20/40 - 20/200 in the better eye;  Blindness: BCVA <20/200 in the better eye |  | 18.2/ 16.6/ 20.3 | 1.9/ 1.4/ 2.2 |  |  |
|  |  |  |  |  |  | |  | |  | | LogMAR E chart, BCVA | | US | Low vision: BCVA <20/40 - 20/200 in the better eye;  Blindness: BCVA <20/200 in the better eye |  | 12.8/ 11.1/ 14.3 | 1.5/ 1.1/ 1.8 |  |  |
| Zhang et al, 2018(18) | 2014 | Hohhot  (Inner Mongolia) | Urban+ rural | 3985 | 42.8 | | 88.6 | | ≥50 | | RAAB (LogMAR) tumbling E chart, PVA | | WHO | MVI: PVA <6/18 to ≥6/60;  SVI: PVA <6/60 to ≥3/60;  Low vision: include both MVI and SVI categories;  Blindness: PVA <3/60 | MVI: 4.8/ 4.4/ 5.2;  SVI: 0.8/ 0.6/ 1.0 | 5.7/ 5.0/  6.2 | 1.1/ 0.8/ 1.3 | MVI: uncorrected refractive error, cataract, posterior segment disease;  SVI: uncorrected refractive error, cataract, posterior segment disease;  Blindness: cataract, posterior segment disease, corneal scar |  |
| Yang et al, 2016(19) | Na | Xishuangbanna Autonomous Prefecture (Yunnan) | Rural | 2163 | 36.8 | | 80.5 | | ≥50 | | logMAR chart with tumbling-E optotypes,  PVA | | WHO | VI: VA of less than 20/63 in the better-seeing eye;  Low vision: VA <20/63–20/400 in the better-seeing eye;  Blindness: VA <20/400 in the better-seeing eye | Total: 15.9 | 13.3/ 10.63/ 14.93 | 3.00/ 3.67/ 2.57 | VI: cataract, refractive error, AMD;  Low vision: cataract, refractive error, AMD;  Blindness: cataract, myopic maculopathy, glaucoma | VI: age and gender |
|  |  |  |  |  |  | |  | |  | | logMAR chart with tumbling-E optotypes,  BCVA | | WHO | VI: VA of less than 20/63 in the better-seeing eye;  Low vision: VA <20/63–20/400 in the better-seeing eye;  Blindness: VA <20/400 in the better-seeing eye | Total: 8.0 | 6.70/ 5.06/ 7.72 | 2.10/ 2.78/ 1.76 |  |  |
|  |  |  |  |  |  | |  | |  | | logMAR chart with tumbling-E optotypes,  PVA | | US | Low vision: VA of less than 20/40 but more than 20/200 in the better-seeing eye;  Legal blindness: VA of not more than 20/200 |  | Total: 23.8 | Total: 7.4 |  |  |
|  |  |  |  |  |  | |  | |  | | logMAR chart with tumbling-E optotypes,  BCVA | | US | Low vision: VA of less than 20/40 but more than 20/200 in the better-seeing eye;  Legal blindness: VA of not more than 20/200 |  | Total: 13.7 | Total: 4.2 |  |  |
| Meng et al, 2021(20) | 2015-2017 | Tianjin | Urban+ rural | 12233 | 50.95 | | 96.83 | | 0-121 | | ETDRS E-chart, BCVA | | WHO | Bilateral VI: BCVA < 20/63 in the better eye;  Bilateral low vision: BCVA < 20/63 to ≥ 20/400 in the better eye;  Bilateral blindness: BCVA < 20/400 in the better eye | 1.86/1.83/3.27 | 1.76/ 1.73/ 3.08 | 0.11/0.10/ 0.18 | Vision loss: cataract, refractive error/amblyopia, AMD for participants over 50 years;  Blindness: cataract, refractive error/amblyopia and glaucoma for participants over 50 years | VI: female gender, older age and diabetes;  Low vision: diabetes for 50+ years old participants;  Blindness: age |
| Jiachu et al, 2018(21) | 2017 | Kandze Tibetan Autonomous Prefecture (Sichuan) | Rural | 4764 | 43.8 | | 95.3 | | ≥50 | | RAAB Snellen tumbling E chart,  PVA | | WHO | EVI: VA of 6/18 to less than 6/12;  MVI: VA of 6/60 or better and less  than 6/18;  SVI: 3/60 or better and less than 6/60;  Blindness: VA in the better eye of less than 3/60 | EVI: 6.41/ 4.88/ 7.75;  MVI: 4.25/ 4.12/ 4.46;  SVI: 0.77/ 0.64/ 0.88 | Na | 1.59/ 1.29/ 1.88 | EVI: uncorrected refractive error, cataract;  MVI: cataract, macular degeneration, other posterior segment disease;  SVI: cataract, macular degeneration;  Blindness: cataract, other posterior segment disease, macular degeneration, and corneal opacity |  |
| Xiong et al, 2020(22) | 2018 | Qianjiang (Chongqing) | Rural | 2112 | 35 | | 83 | | ≥50 | | Snellen E chart, PVA | | WHO | VI: BCVA worse than 0.3;  Low vision: VA <0.3 to ≥0.05;  Blindness: VA was worse than 0.05 in the better eye | Total: 11.2 | 11.2/ 10.0/ 11.8 | 4.0/ 5.7/ 3.1 | VI: cataract, refractive error, AMD;  Low vision: cataract, refractive error, AMD;  Blindness: cataract, refractive error, myopic maculopathy |  |
|  |  |  |  |  |  | |  | |  | | Snellen E chart, BCVA | | WHO | VI: BCVA worse than 0.3;  Low vision: VA <0.3 to ≥0.05;  Blindness: VA was worse than 0.05 in the better eye | Total: 7.8 | 6.2/ 4.5/  7.1 | 1.8/ 2.8/ 1.2 | VI: cataract, AMD, corneal opacity;  Low vision: cataract, AMD, posterior capsular opacification;  Blindness: cataract, AMD, corneal opacity |  |
|  |  |  |  |  |  | |  | |  | | Snellen E chart, PVA | | US | Low vision: VA <0.5 to >0.01;  Blindness: VA was worse than 0.1 in the better eye |  | Total: 25.5 | Total: 4.9 |  |  |
|  |  |  |  |  |  | |  | |  | | Snellen E chart, BCVA | | US | Low vision: VA <0.5 to >0.01;  Blindness: VA was worse than 0.1 in the better eye |  | Total: 10.0 | Total: 2.5 |  |  |

ETDRS: Early Treatment Diabetic Retinopathy Study; WHO: World Health Organization; US: United States; Na: not applicable; VI, visual impairment; SVI: severe visual impairment; MVI: moderate visual impairment; EVI: early visual impairment; MVI: moderate visual impairment; BCVA: best corrected visual acuity; PVA, presenting visual acuity; AMD: age-related macular degeneration; RAAB: Rapid Assessment of Avoidable Blindness.

**Supplementary Table S2. Detailed characteristics of the main studies on visual impairment incidence in mainland China.**

| **Study** | **Survey year** | **Location** | **Population** | **Male (%)** | **Response rate (%)** | **Age ranges/ Mean age** | **Follow-up years** | **Instrument and measurement** | **Criteria** | **Definition** | **Incidence (%)**  **Total/Male/Female** | | | **Main cause** | **Risk factors** |
| --- | --- | --- | --- | --- | --- | --- | --- | --- | --- | --- | --- | --- | --- | --- | --- |
|  |  |  |  |  |  |  |  |  |  |  | **VI** | **Low vision** | **Blindness** |  |  |
| You et al, 2011(23) | 2001 (2006) | Beijing | 3249 | 43.6 | 73.2 | 40-84 | 5 | Snellen chart, PVA | WHO | Low vision: BCVA worse than 20/60 to 20/400 in the better-seeing eye;  Blindness: BCVA worse than 20/400 in the better-seeing eye | Na | 1.7/  0.9/  2.3 | Na |  |  |
|  |  |  |  |  |  |  |  | Snellen chart, BCVA | WHO | Low vision: BCVA worse than 20/60 to 20/400 in the better-seeing eye;  Blindness: BCVA worse than 20/400 in the better-seeing eye | Na | 0.5/  0.1/  0.8 | 0.1/ 0.1/ 0.1 | Low vision: cataract, glaucoma, degenerative myopia |  |
|  |  |  |  |  |  |  |  | Snellen chart, PVA | US | Low vision: BCVA worse than 20/40 to 20/200 in the better-seeing eye;  Blindness: BCVA worse than 20/200 in the better-seeing eye | Na | 3.8/ 3.3/  4.2 | Na |  |  |
|  |  |  |  |  |  |  |  | Snellen chart, BCVA | US | Low vision: BCVA worse than 20/40 to 20/200 in the better-seeing eye;  Blindness: BCVA worse than 20/200 in the better-seeing eye | Na | 1.1/  0.8/  1.3 | 0.1/ 0.1/ 0.1 |  |  |
| Wang et al, 2013(24) | 2003 (2009) | Liwan (Gaungzhou) | 924 | 42.6 | 75.0 | ≥50/  63.4 ± 9.0 | 5 | ETDRS visual chart, PVA | WHO | VI: BCVA ≥ 20/60 in both eyes at baseline, which decreased to <20/60 to 20/400 in the better-seeing eye at follow-up;  Blindness: BCVA ≥ 20/400 in both eyes at baseline, which decreased to <20/400 in the better-seeing eye at follow-up | Total:12.4 | Na | Total: 0.33 | VI: refractive error, cataract, and AMD | VI: older age, less education, and poorer PVA at baseline in the worse-seeing eye |
|  |  |  |  |  |  |  |  | ETDRS visual chart, BCVA | WHO | VI: BCVA ≥ 20/60 in both eyes at baseline, which decreased to <20/60 to 20/400 in the better-seeing eye at follow-up;  Blindness: BCVA ≥ 20/400 in both eyes at baseline, which decreased to <20/400 in the better-seeing eye at follow-up | Total: 5.38 | Na | Total: 0.33 | VI: cataract, myopic macular degeneration, glaucoma, and diabetic macular edema | VI: older age, less education, and poorer PVA at baseline in the worse-seeing eye |
|  |  |  |  |  |  |  |  | ETDRS visual chart, PVA | US | VI: BCVA ≥ 20/40 in both eyes at baseline, which decreased to <20/40 to 20/200 in the better-seeing eye at follow-up;  Blindness: BCVA ≥ 20/200 in both eyes at baseline, which decreased to <20/200 in the better-seeing eye at follow-up | Total: 20.6 | Na | Total: 1.77 | VI: refractive error, cataract, and AMD |  |
|  |  |  |  |  |  |  |  | ETDRS visual chart, BCVA | US | VI: BCVA ≥ 20/40 in both eyes at baseline, which decreased to <20/40 to 20/200 in the better-seeing eye at follow-up;  Blindness: BCVA ≥ 20/200 in both eyes at baseline, which decreased to <20/200 in the better-seeing eye at follow-up | Total: 9.85 | Na | Total: 1.42 | VI: cataract, myopic macular degeneration, and AMD |  |
| Wang et al, 2021(25) | 2003 (2013-2014) | Liwan (Gaungzhou) | 1399 | 43.6 | 75.4 | 65.3 ± 9.93 | 10 | ETDRS visual chart, PVA |  | VI: PVA < 20/40 in the better-seeing eye | Total: 22.9 | Na | Na |  |  |
|  |  |  |  |  |  |  |  | ETDRS visual chart, BCVA |  | VI: BCVA < 20/40 in the better-seeing eye | Total: 9.43 | Na | Na |  |  |
| Hu et al, 2021(26) | 2006 (2012) | Handan (Hebei) | 5333 | 44.6 | Na | ≥50/  51.4 ± 11.1 | 6 | LogMAR E chart, PVA | WHO | Low vision: VA ≥20/60 in both eyes at baseline, which decreased to <20/60 but ≥20/400 in the better-seeing eye at follow-up;  Blindness: VA ≥20/400 in both eyes at baseline, which decreased to <20/400 in the better-seeing eye at follow-up | Na | 5.2/ 5.3/  8.8 | 0.5/ 0.7/ 0.8 | Low vision: cataract, refractive error, myopic retinopathy, AMD, glaucoma, DR, corneal disease;  Blindness: cataract, myopic retinopathy, refractive error, corneal disease, AMD, glaucoma | Low vision: age, education level, BMI, diabetes; Blindness: diabetes |
|  |  |  |  |  |  |  |  | LogMAR E chart, BCVA | WHO | Low vision: VA ≥20/60 in both eyes at baseline, which decreased to <20/60 but ≥20/400 in the better-seeing eye at follow-up;  Blindness: VA ≥20/400 in both eyes at baseline, which decreased to <20/400 in the better-seeing eye at follow-up | Na | 0.8/ 1.0/  2.0 | 0.1/ 0.1/ 0.1 | Low vision: cataract, myopic retinopathy, refractive error, glaucoma, DR, AMD, corneal disease;  Blindness: cataract | Low vision: age, BMI |
| Han et al, 2019(27) | 2008 (2014) | Yuexiu (Guangzhou) | 1369 | 45.1 | 78.5 | ≥35/  52.3 ± 11.6 | 6 | ETDRS visual chart, PVA | WHO | VI: binocular PVA <20/60 to 20/400;  Blindness: binocular PVA <20/400 | Total: 8.33 | Na | Na | VI: undercorrected refractive error | VI: older age, female, lower education level, more myopic spherical equivalent and worse PVA at baseline |
|  |  |  |  |  |  |  |  | ETDRS visual chart, PVA | US | VI: binocular PVA <20/40 to 20/200;  Blindness: binocular PVA <20/200 | Total: 12.2 | Na | Na |  | VI: older age, lower education level, more myopic spherical equivalent and worse PVA at baseline |

ETDRS: Early Treatment Diabetic Retinopathy Study; WHO: World Health Organization; US: United States; Na: not applicable; VI, visual impairment; BCVA: best corrected visual acuity; PVA, presenting visual acuity; AMD: age-related macular degeneration; DR: diabetic retinopathy

**Reference:**

1. Zhao J, Jia L, Sui R, and Ellwein LB. Prevalence of blindness and cataract surgery in Shunyi County, China. *Am J Ophthalmol*. (1998) 126: 506-14. 10.1016/s0002-9394(98)00275-x.

2. Li S, Xu J, He M, Wu K, Munoz SR, and Ellwein LB. A survey of blindness and cataract surgery in Doumen County, China. *Ophthalmology*. (1999) 106: 1602-08. 10.1016/S0161-6420(99)90459-1.

3. Xu L, Wang Y, Li Y, Wang Y, Cui T, Li J, et al. Causes of blindness and visual impairment in urban and rural areas in Beijing: the Beijing Eye Study. *Ophthalmology*. (2006) 113: 1134.e1-34.11. 10.1016/j.ophtha.2006.01.035.

4. Huang S, Zheng Y, Foster PJ, Huang W, and He M. Prevalence and causes of visual impairment in Chinese adults in urban southern China. *Arch Ophthalmol*. (2009) 127: 1362-67. 10.1001/archophthalmol.2009.138.

5. Wu M, Yip JLY, and Kuper H. Rapid assessment of avoidable blindness in Kunming, china. *Ophthalmology*. (2008) 115: 969-74. 10.1016/j.ophtha.2007.08.002.

6. Zhao J, Ellwein LB, Cui H, Ge J, Guan H, Lv J, et al. Prevalence of vision impairment in older adults in rural China: the China Nine-Province Survey. *Ophthalmology*. (2010) 117: 409-16. 10.1016/j.ophtha.2009.11.023.

7. Li T, Du L, and Du L. Prevalence and Causes of Visual Impairment and Blindness in Shanxi Province, China. *Ophthalmic Epidemiol*. (2015) 22: 239-45. 10.3109/09286586.2015.1009119.

8. Guo C, Wang Z, He P, Chen G, and Zheng X. Prevalence, Causes and Social Factors of Visual Impairment among Chinese Adults: Based on a National Survey. *Int J Environ Res Public Health*. (2017) 14: 1034. 10.3390/ijerph14091034.

9. Liang YB, Friedman DS, Wong TY, Zhan SY, Sun LP, Wang JJ, et al. Prevalence and causes of low vision and blindness in a rural chinese adult population: the Handan Eye Study. *Ophthalmology*. (2008) 115: 1965-72. 10.1016/j.ophtha.2008.05.030.

10. Song W, Sun X, Shao Z, Zhou X, Kang Y, Sui H, et al. Prevalence and causes of visual impairment in a rural North-east China adult population: a population-based survey in Bin County, Harbin. *Acta Ophthalmol*. (2010) 88: 669-74. 10.1111/j.1755-3768.2009.01768.x.

11. Xiao B, Kuper H, Guan C, Bailey K, and Limburg H. Rapid assessment of avoidable blindness in three counties, Jiangxi Province, China. *Br J Ophthalmol*. (2010) 94: 1437-42. 10.1136/bjo.2009.165308.

12. Li Z, Cui H, Liu P, Zhang L, Yang H, and Zhang L. Prevalence and causes of blindness and visual impairment among the elderly in rural southern Harbin, China. *Ophthalmic Epidemiol*. (2008) 15: 334-38. 10.1080/09286580802227386.

13. Cheng F, Shan L, Song W, Fan P, and Yuan H. Distance- and near-visual impairment in rural Chinese adults in Kailu, Inner Mongolia. *Acta Ophthalmol*. (2016) 94: 407-13. 10.1111/aos.12808.

14. Li J, Zhong H, Cai N, Luo T, Li J, Su X, et al. The prevalence and causes of visual impairment in an elderly Chinese Bai ethnic rural population: the Yunnan minority eye study. *Invest Ophthalmol Vis Sci*. (2012) 53: 4498-504. 10.1167/iovs.12-9429.

15. Li EY, Liu Y, Zhan X, Liang YB, Zhang X, Zheng C, et al. Prevalence of blindness and outcomes of cataract surgery in Hainan Province in South China. *Ophthalmology*. (2013) 120: 2176-83. 10.1016/j.ophtha.2013.04.003.

16. Zhang X, Li EY, Leung CK-S, Musch DC, Tang X, Zheng C, et al. Prevalence of visual impairment and outcomes of cataract surgery in Chaonan, South China. *PLoS One*. (2017) 12: e0180769. 10.1371/journal.pone.0180769.

17. Tang Y, Wang X, Wang J, Huang W, Gao Y, Luo Y, et al. Prevalence and Causes of Visual Impairment in a Chinese Adult Population: The Taizhou Eye Study. *Ophthalmology*. (2015) 122: 1480-88. 10.1016/j.ophtha.2015.03.022.

18. Zhang G, Tham Y-C, Gong H, Ren F, Morige J, Dai W, et al. Blindness, low vision and cataract surgery outcome among adults in Hohhot of Inner Mongolia: a Rapid Assessment of Avoidable Blindness (RAAB) study. *Br J Ophthalmol*. (2018) 102: 1653-57. 10.1136/bjophthalmol-2017-311633.

19. Yang W-Y, Li J, Zhao C-H, Qian D-J, Niu Z, Shen W, et al. Population-based assessment of visual impairment among ethnic Dai adults in a rural community in China. *Sci Rep*. (2016) 6: 22590. 10.1038/srep22590.

20. Meng X, Zhou W, Sun Z, Han Q, Zhang J, Zhang H, et al. Prevalence and causes of bilateral visual impairment in rural areas of Tianjin, China - The Tianjin Eye Study. *Acta Ophthalmol*. (2021) 99: e136-e43. 10.1111/aos.14523.

21. Jiachu D, Jiang F, Luo L, Zheng H, Duo J, Yang J, et al. Blindness and eye disease in a Tibetan region of China: findings from a Rapid Assessment of Avoidable Blindness survey. *BMJ Open Ophthalmol*. (2018) 3: e000209. 10.1136/bmjophth-2018-000209.

22. Xiong X, Liu D, Liu S, Wu M, Zhan B, Wang H, et al. The prevalence and causes of visual impairment among ethnic Tujia adults in a rural community in China. *Medicine (Baltimore)*. (2020) 99: e22464. 10.1097/MD.0000000000022464.

23. You QS, Xu L, Yang H, Wang YX, and Jonas JB. Five-year incidence of visual impairment and blindness in adult Chinese the Beijing Eye Study. *Ophthalmology*. (2011) 118: 1069-75. 10.1016/j.ophtha.2010.09.032.

24. Wang L, Huang W, He M, Zheng Y, Huang S, Liu B, et al. Causes and five-year incidence of blindness and visual impairment in urban Southern China: the Liwan Eye Study. *Invest Ophthalmol Vis Sci*. (2013) 54: 4117-21. 10.1167/iovs.13-11911.

25. Wang L, Zhu Z, Scheetz J, and He M. Visual impairment and ten-year mortality: the Liwan Eye Study. *Eye (Lond)*. (2021) 35: 2173-79. 10.1038/s41433-020-01226-x.

26. Hu A, Gu SZ, Friedman DS, Cao K, and Wang N. Six-Year Incidence and Causes of Low Vision and Blindness in a Rural Chinese Adult Population: The Handan Eye Study. *Ophthalmic Epidemiol*. (2021) 28: 160-68. 10.1080/09286586.2020.1795886.

27. Han X, Liao C, Liu C, Lee PY, Zhang J, Keel S, et al. Incidence and correction of vision impairment among elderly population in southern urban China. *Clin Exp Ophthalmol*. (2019) 47: 439-44. 10.1111/ceo.13431.
